# The Efficacy of a Novel Hybrid Brace in the Treatment of Adolescent Idiopathic Scoliosis: A Prospective Case-Series Study

**DOI:** 10.3390/children12030328

**Published:** 2025-03-05

**Authors:** Hyoungmin Kim, Sam Yeol Chang, Bong Soon Chang, Jun Yeop Lee, Seonpyo Jang, Sung Taeck Kim

**Affiliations:** Department of Orthopaedic Surgery, Seoul National University Hospital, 101 Daehangno, Jongrogu, Seoul 03080, Republic of Korea; hmkim21@gmail.com (H.K.); bschang@snu.ac.kr (B.S.C.); lssjy1011@gmail.com (J.Y.L.); sshs1010@gmail.com (S.J.); kstbest0915@naver.com (S.T.K.)

**Keywords:** adolescent idiopathic scoliosis, brace, Lenke classification, Risser sign

## Abstract

**Background/Objectives**: Bracing is an effective treatment for preventing curve progression in skeletally immature adolescent idiopathic scoliosis (AIS) patients. A novel hybrid brace has been developed to overcome the limitations of conventional rigid and soft braces. This study aimed to evaluate the clinical efficacy of the novel hybrid brace. **Methods**: We enrolled AIS patients who were candidates for brace treatment: aged 10–18 years, with a coronal Cobb angle of 20–45° and a Risser stage of 0–2. The primary outcome was the rate of successful brace treatment, defined as meeting all three criteria: (1) less than 5° of progression in the Cobb angle during follow-up, (2) less than 45° of Cobb angle at the final follow-up, and (3) avoidance of surgical treatment. **Results**: A total of 24 patients (1 male, 23 female) with a mean age of 12.2 ± 1.2 years were included in this study. At the initiation of bracing, the major curve had a mean Cobb angle of 34.5 ± 6.3° and an in-brace correction (IBC) rate of 41.5 ± 16.0%. The hybrid brace demonstrated a success rate of 91.7% (22/24) during a mean follow-up period of 22.1 ± 6.4 months. After brace treatment, seven (29.2%) patients showed an improvement of more than 5° in their Cobb angle. When compared to a matched control from a retrospective cohort, the hybrid brace demonstrated a greater success rate (91.7% vs. 83.3%) and a higher proportion of patients with an improved curve (29.2% vs. 12.5%), although statistically insignificant. **Conclusions**: A novel hybrid brace was effective in preventing curve progression in skeletally immature patients with AIS.

## 1. Introduction

Adolescent idiopathic scoliosis (AIS) is a common disorder affecting 0.5% to 5.0% of adolescents, placing a significant socioeconomic burden on society [1,2]. Bracing is widely accepted as an effective non-operative treatment for preventing curve progression in skeletally immature AIS patients [3,4,5]. Although some studies reported superior clinical outcomes of full-time braces over part-time braces, there is still a lack of evidence in the literature on which brace is the most appropriate among various types and the optimal daily wearing time.

Various braces have been introduced for AIS treatment. Although rigid braces have long been the standard, they have several limitations, such as skin issues and psychological stress [2,6]. In response, soft braces were developed to address these problems. However, previous studies have shown that soft braces tend to have lower success rates compared to rigid braces [7,8].

**Figure 1:**
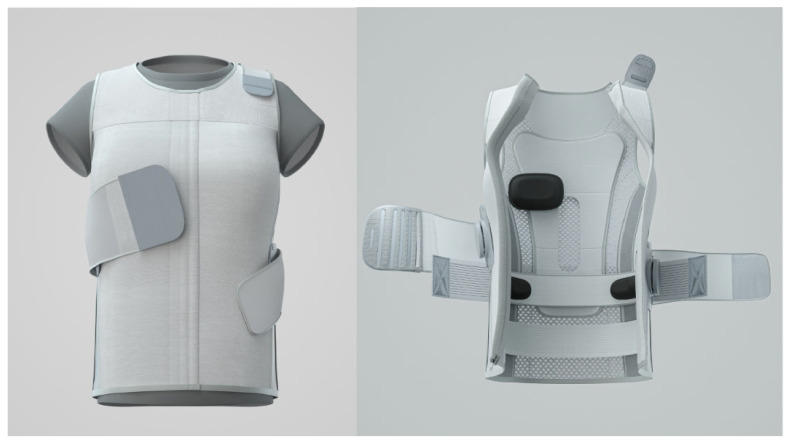
A novel hybrid brace utilizing three-point pressure provided by pressure pads and bands attached to a vest. An additional hump pad further facilitates derotation of the scoliosis.

A novel hybrid brace has been designed to overcome the limitations of both rigid and soft braces, offering an effective and more comfortable treatment option. The hybrid brace corrects scoliosis by applying three-point pressure through pressure pads and elastic bands attached to a vest (Figure 1). According to the classification proposed by Negrini S et al., the current hybrid brace is an elastic thoracolumbosacral orthosis with a primary action of three points [9]. Theoretically, the hybrid brace is less noticeable and stressful for young patients compared to rigid braces, while being more effective in scoliosis correction than soft braces. However, there is limited evidence regarding the clinical efficacy of hybrid braces. Thus, this prospective case series study aimed to evaluate the effectiveness of the hybrid brace in preventing curve progression in AIS patients. We postulated that the hybrid brace will show comparable efficacy in preventing curve progression in AIS patients compared to conventional braces.

## 2. Materials and Methods

### 2.1. Study Design and Participants

This prospective case series study enrolled patients with AIS who were candidates for brace treatment. The indications for brace treatment were based on the guidelines of the Scoliosis Research Society (SRS) and the International Society on Scoliosis Orthopaedic and Rehabilitation Treatment and included the following: age 10–18 years, a curve with a coronal Cobb angle of 20–45°, and a Risser stage of 0–2 [10,11]. Only patients who were willing to wear braces for at least 8 h a day at night were enrolled.

Patients with a history of previous brace treatment or spine surgery, congenital spinal deformities, or comorbidities associated with spinal deformities (such as neuromuscular diseases, skeletal dysplasia, or a history of sternotomy) were excluded. Only patients who were followed for at least 12 months after starting brace treatment were included in the analysis. The study was approved by the Institutional Review Board of our institution (IRB No.: 2111-078-1272), and all participants and their guardians provided written informed consent.

### 2.2. Hybrid Brace Treatment and Follow-Up

**Figure 2:**
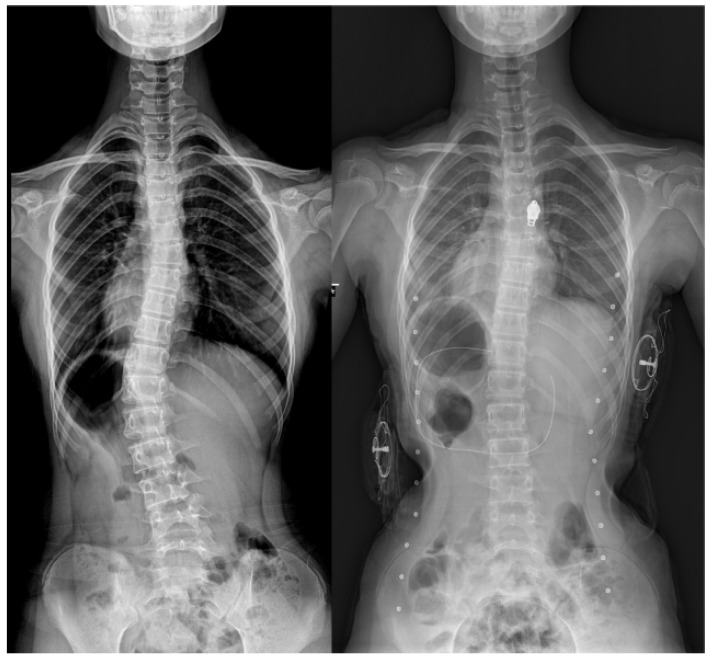
In-brace correction of scoliosis using the hybrid brace.

A novel hybrid brace was prescribed for all participants (Figure 1). This hybrid brace corrects scoliosis using the “three-point pressure” mechanism [12]. Unlike rigid braces, the hybrid brace applies three-point pressure via pressure pads and bands attached to the vest. These pressure pads provide three-point pressure on (1) the apex of the major curve, (2) the contralateral shoulder, and (3) contralateral pelvis to aid the straightening of scoliosis. An additional hump pad placed on the thoracic or lumbar hump, identified using the Adams forward bending test, further facilitates derotation of the scoliosis. The vest-type feature of the current hybrid brace allows the location of a pressure pad at the shoulder opposite to the curve apex, and an additional hump pad, which differentiates it from traditional braces. Each hybrid brace was customized by adjusting the location of pressure pads and hump pads to maximize in-brace correction (Figure 2).

All participants were instructed to wear the hybrid brace at night, including during sleep, for more than 8 h a day. Patients who could not adhere to this instruction were excluded from the study. We did not provide standardized rehabilitation during the study period to all participants, and they were allowed to receive standard care in their respective regions. All patients visited the outpatient clinic every 3 months for evaluation and adherence check-ups. The brace was discontinued when patients reached ulnar grade 8 (U8) in the distal radioulnar (DRU) classification [13,14] or when surgical treatment was recommended due to curve progression despite brace treatment.

### 2.3. Data Collection and Outcome Assessment

Demographic data, skeletal maturity information, and scoliosis type were collected at enrollment. Skeletal maturity was assessed using the Risser grade and DRU classification, and curve type was classified using the Lenke classification. The coronal Cobb angle of the major curve, and the location and rotation of the apical vertebra (AV) identified using the Nash–Moe method, were assessed using standing whole spine anteroposterior (AP) X-rays. After fitting the customized hybrid brace, a whole spine AP X-ray was taken, and in-brace correction (IBC) was calculated using the following formula: IBC (%) = 100% × (pre-brace standing Cobb − post-brace standing Cobb)/pre-brace standing Cobb.

Patient-reported outcomes were assessed using the Scoliosis Research Society-22 (SRS-22) questionnaire at the start of brace treatment. During follow-up, whole spine radiographs were taken every 3 months to assess curve progression, and skeletal maturity was assessed annually using the Risser grade and DRU classification. All radiographs were randomized and anonymized before being evaluated by blinded observers.

The primary outcome was the rate of successful brace treatment, defined as meeting all three criteria: (1) less than 5° progression in Cobb angle during follow-up, (2) final Cobb angle < 45°, and (3) avoidance of surgical treatment. The secondary outcomes were IBC at brace initiation, changes in Cobb angle, and SRS-22 scores at 12 months post-brace treatment. Patients were classified into three groups based on changes in Cobb angle: (1) improvement (more than 5°), (2) stationary, and (3) progression (more than 5°).

### 2.4. Statistical Analysis

Primary and secondary outcomes were compared with a matched control group of patients who received treatment with a night-time rigid brace (Charleston-type). The control group was matched for age, sex, Risser grade, and curve types (Lenke classification) in a 1:1 ratio using random number generation. Statistical tests included Fisher’s exact and Pearson’s chi-squared tests for categorical data, Mann-Whitney U and independent *t*-tests for continuous variables, and the Wilcoxon signed-rank test to assess changes from baseline. The Statistical Package for the Social Sciences software version 28.0 (IBM Corp., Armonk, NY, USA) was used for analysis, with statistical significance set at *p* < 0.05.

## 3. Results

A total of 31 patients were initially enrolled in this prospective study. However, 7 patients withdrew during the follow-up period. Consequently, 24 patients (1 male, 23 female) with a mean age of 12.2 ± 1.2 years were included in the final analysis. Regarding skeletal maturity, the mean Risser grade was 1.9 ± 1.6, and the mean ulnar grade of the DRU classification was 6.5 ± 1.0 at the start of brace treatment. 

Curve types were classified based on the topographic and Lenke classifications. Among 24 patients, 8 (33.3%) patients had thoracic-type scoliosis (Lenke 1A: 4, 1B: 2, 1C: 2), 13 (54.2%) had lumbar-type scoliosis (Lenke 5C), and 3 (12.5%) had S-shaped scoliosis (Lenke 2B: 2, Lenke 6C: 1). The major curve, with a mean Cobb angle of 34.5 ± 6.3° before brace application, was corrected to 20.4 ± 7.6° when the hybrid brace was initially applied, yielding an in-brace correction (IBC) rate of 41.5 ± 16.0%.

Regarding the primary outcome, treatment with the hybrid brace showed a success rate of 91.7% (22/24) during a mean follow-up period of 22.1 ± 6.4 months. However, in the worst-case scenario, where we assume all seven dropped-out patients have failed to achieve satisfactory clinical results, the success rate is 71.0% (22/31). As for the unsuccessful group, two patients (8.3%), both with Lenke 1A curves and an ulnar grade of U6 in the DRU classification at the start of brace treatment, showed unsuccessful results due to major curve progression exceeding 5° during follow-up. One patient, whose major curve Cobb angle was 44° at the start, underwent corrective surgery after the curve progressed beyond 50° at 12 months post-brace.

**Figure 3:**
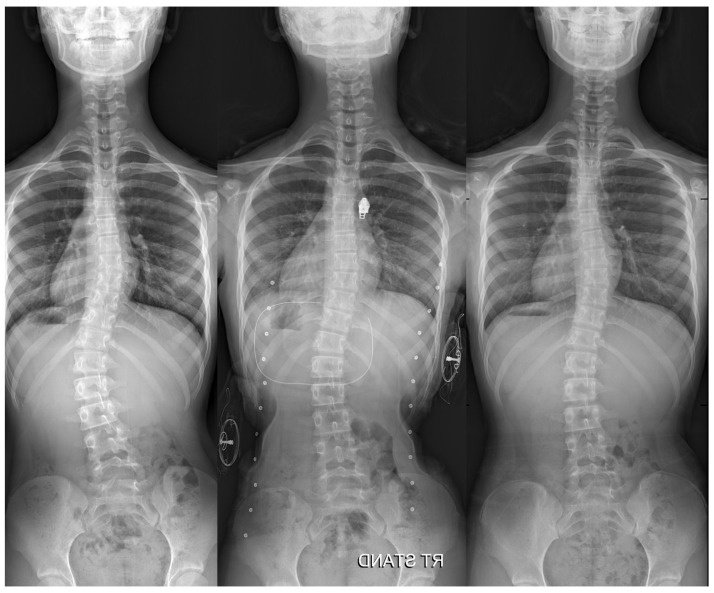
A case of a 12-year-old female patient treated with a hybrid brace. The Cobb angle of the lumbar curve improved from 30° at the brace initiation (Risser grade 1) to 21° at the final follow-up (Risser grade 4).

The final Cobb angle of the major curve was 31.6 ± 8.6° for all patients, with a mean change of −3.0 ± 4.6° from the initiation of brace treatment. When patients were grouped based on the change in Cobb angle, seven (29.2%) showed improvement (>5°), fifteen (62.5%) remained stationary, and two (8.3%) experienced progression (>5°). Table 1 summarizes the distribution of these groups, stratified by skeletal maturity, Lenke curve type, curve magnitude (Cobb angle), and apical vertebra rotation (Nash–Moe) at brace initiation. All seven patients in the improved group had a major Cobb angle of less than 35° at brace initiation, with a mean angle of 29.2° (Figure 3).

Table 2 summarizes changes in the SRS-22 questionnaire scores. For both the improved and stationary groups, all SRS-22 domains showed no statistically significant changes from baseline to 12 months post-brace treatment. Although the satisfaction score at 12 months was higher in the improved group compared to the stationary group, the difference was not statistically significant (2.1 ± 0.5 vs. 1.9 ± 1.0, *p* = 0.769). No complications related to brace treatment, such as brace sores, were reported during the study period.

We generated a 1:1 matched control group (n = 24) with the same age, sex, Risser grade, and Lenke curve type distributions extracted from a retrospective cohort of 251 patients treated with a night-time Charleston rigid brace. When compared to the control group, the hybrid brace demonstrated a higher success rate (91.7% vs. 83.3%) and a greater proportion of patients with an improved major Cobb angle (>5°) (29.2% vs. 12.5%). However, these differences were not statistically significant (Table 3). The IBC was significantly greater in the rigid brace group (hybrid: 41.5% vs. rigid: 61.1%, *p* = 0.001).

## 4. Discussion

The novel hybrid brace evaluated in this prospective study achieved a success rate of 91.7%. Notably, the two patients with unsuccessful outcomes had Lenke 1A curves with Cobb angles exceeding 40° at the start of treatment. All patients with an initial Cobb angle of less than 40° achieved successful outcomes at the final follow-up.

The success rate of the hybrid brace is comparable to similar braces reported in the literature. For instance, the Providence brace, a rigid night-time brace, has shown success rates ranging from 52% to 89% in previous studies [15,16,17,18], while SpineCor, a typical soft brace, reported success rates between 59.4% and 73% [8,19,20]. Compared to these braces, the hybrid brace in this series showed clinical effectiveness in preventing curve progression in skeletally immature patients with AIS.

Grouping patients based on changes in Cobb angle revealed that seven patients experienced significant improvement (>5°). Previous studies have identified multiple factors associated with a favorable outcome after brace treatment, such as a skeletal maturity greater than Risser grade 1 and a curve apex caudal to T10 [15]. However, our crosstab analysis showed that no factor was significantly associated with the improvement of curve magnitude after brace treatment, including skeletal maturity, curve type, and IBC. However, trends indicated that patients with advanced skeletal maturity (>U7) and major lumbar region curves (Lenke 5 and 6) were more likely to experience curve improvement (Table 1).

Additionally, prior studies have demonstrated that patients with smaller curves tend to have better outcomes. For instance, Simony et al. reported success rates of 93% for Cobb angles of 30–39° and 77% for angles of 40–45° using the Providence brace [18]. Similarly, all seven patients in the improved group had a Cobb angle of less than 35° at the brace-initiation in our study. Therefore, it should be noted that patients with milder curves can benefit more from treatment with the novel hybrid brace.

When all 24 patients treated with a novel hybrid brace were compared to the matched control group, the hybrid group showed a greater success rate (91.7% vs. 83.3%) and a higher proportion of patients with an improved major Cobb angle of more than 5° (29.2% vs. 12.5%), although the differences were not statistically significant. However, it should be noted that the control group had a significantly longer follow-up period than the hybrid group. This may have led to more patients with an increase in Cobb angle for more than 5° during the follow-up.

Previous studies have emphasized the importance of IBC as a predictor of successful brace treatment [16]. Some authors have advocated a threshold IBC of 25% for successful brace treatment [21]. The hybrid brace in our study achieved a mean IBC of 41.5%, exceeding this threshold and comparable to other braces [22]. However, it is worth noting that rigid braces generally show a greater IBC in the literature [7,18]. In our study, the IBC of the control group (Charleston night-time brace) was significantly greater than the hybrid group (61.1% vs. 41.5%, *p* = 0.001). Among the hybrid brace patients, those with progressive curves had an IBC below 40%, highlighting the need for further investigation into the implications of low IBC in hybrid brace treatment.

In this study, the patients were instructed to wear the hybrid brace only at nighttime. Although there are controversies on the appropriate brace-wearing time in the literature, recent studies have shown favorable clinical outcomes of nighttime bracing in AIS patients [2,7]. In addition, considering that a full-time brace, including time at school, may induce more psychological stress in sensitive adolescents, we chose nighttime bracing in our study. However, nighttime bracing with the current hybrid brace was different from a traditional rigid brace, such as the Charleston brace, in that it does not overcorrect the scoliosis. Despite the lack of overcorrection during the brace-wearing time limited only to nights, the current hybrid brace showed comparable efficacy in our study.

As for the patient-reported outcomes, we utilized SRS-22 and found that the scores did not significantly change before and after brace treatment (Table 2). More interestingly, the SRS-22 scores of the total cohort were relatively low compared to the normal values reported in the literature [23]. Because the SRS-22 scores were low from the baseline even before the start of brace treatment, such low SRS-22 scores seem to be a characteristic of patients visiting our institution rather than the effect of brace treatment. However, deter-mining the cause for such low SRS-22 scores in our patients is beyond the scope of this study.

This study has several limitations. First, the sample size was relatively small, limiting the statistical power to identify factors associated with successful outcomes. Second, although the patients were followed until a mean Risser grade of 3.9, the mean age of the total cohort at the final follow-up was 14.0 years old, suggesting that the patients may have had remaining growth potential. Therefore, a longer follow-up might reveal lower success rates than reported in this study. Third, the control group had a longer follow-up period, potentially biasing comparisons. Fourth, during the study period, we did not pro-vide standardized rehabilitation or exercise programs, which are important treatment modalities for AIS. Future studies should implement and control rehabilitation or exercise programs to better clarify the clinical effectiveness of bracing. Fifth, this study’s drop-out rate was high, suggesting a high attrition bias. In the worst-case scenario where all drop-out patients had failed, the success rate becomes 71.0% (22/31). Finally, no objective assessment of brace wear time or compliance was conducted despite their importance in determining treatment outcomes. Future studies should compare the compliance between the two types of braces with objective measurements of brace-wearing time. Despite these limitations, this prospective case series provides valuable insights into the clinical out-comes of the novel hybrid brace.

## 5. Conclusions

The novel hybrid brace in this prospective case series demonstrated effectiveness in preventing curve progression in skeletally immature patients with AIS.

## Figures and Tables

**Table 1:** The outcomes of hybrid brace treatment stratified by the baseline patient characteristics.

		Improved	Stationary	Progressed	*p*-Value *
All patients (n = 24)		7 (29.2%)	15 (62.5)	2 (8.3%)	
DRU classification	Ulnar grade < 7 (n = 14)	2 (14.3%)	10 (71.4%)	2 (14.3%)	0.172
	Ulnar grade ≥ 7 (n = 10)	5 (50.0%)	5 (50.0%)	0	
Curve type	Lenke 1, 2 (n = 10)	2 (20.0%)	6 (60.0%)	2 (20.0%)	1.000
	Lenke 5, 6 (n = 14)	5 (35.7%)	9 (64.3%)	0	
Curve magnitude (pre-brace)	20–29° (n = 7)	3 (42.9%)	4 (57.1%)	0	0.423
	30–39° (n = 11)	2 (18.2%)	9 (81.8%)	0	
	40–45° (n = 6)	2 (33.3%)	2 (33.3%)	2 (33.3%)	
In-brace correction	<40% (n = 10)	3 (30.0%)	5 (50.0%)	2 (20.0%)	1.000
	≥40% (n = 14)	4 (28.6%)	10 (71.4%)	0	
Apical vertebra rotation	Nash–Moe < 2 (n = 19)	5 (26.3%)	13 (68.4%)	1 (5.3%)	0.565
	Nash–Moe ≥ 2 (n = 5)	2 (40.0%)	2 (40.0%)	1 (20.0%)	

* *p*-values from determining the difference in the proportion of patients with an improved Cobb angle.

**Table 2:** Change in patient-reported outcomes (SRS-22 questionnaire) at baseline and 12 months after brace treatment.

	Improved Group (n = 7)			Stationary Group (n = 15)		
	Baseline	12 Months	*p*-Value *	Baseline	12 Months	*p*-Value *
SRS-22 Mean	2.6 ± 0.3	2.7 ± 0.2	0.938	2.6 ± 0.2	2.8 ± 0.6	0.223
SRS-22 Function	2.5 ± 0.3	2.5 ± 0.2	1.000	2.8 ± 0.3	2.8 ± 0.6	1.000
SRS-22 Pain	3.2 ± 0.2	3.1 ± 0.1	0.750	3.2 ± 0.1	3.2 ± 0.5	0.231
SRS-22 Self-image	2.5 ± 0.8	2.6 ± 0.8	0.797	2.1 ± 0.5	2.5 ± 0.9	0.116
SRS-22 Mental health	2.3 ± 0.4	2.3 ± 0.2	0.875	2.2 ± 0.3	2.3 ± 0.7	0.902

* *p*-values from comparing scores at baseline and 12 months after brace treatment.

**Table 3:** Comparison of treatment outcomes between hybrid and rigid brace (matched control).

	Hybrid Brace (n = 24)	Rigid Brace (n = 24)	Cohen’s d	*p*-Value
Initial Cobb angle (°)	34.5 ± 6.3	32.3 ± 5.2	0.39	0.189
In-brace correction (%)	41.5 ± 16.0	61.1 ± 19.2	1.11	0.001
Final Cobb angle (°)	31.6 ± 8.6	32.6 ± 7.6	0.12	0.678
Success rate (%)	91.7 (22/24)	83.3 (20/24)	NA	0.666
Improved group (%)	29.2 (7/24)	12.5 (3/24)	NA	0.287
Final Risser grade	3.9 ± 1.1	3.8 ± 0.7	0.18	0.541
Follow-up (months)	21.1 ± 6.4	31.6 ± 15.5	0.89	0.003

## Data Availability

Data underlying this article cannot be shared publicly because of the privacy of individuals who participated in the study. The data may be shared with the corresponding authors upon reasonable request.

## Abbreviations

The following abbreviations are used in this manuscript:

AIS	Adolescent idiopathic scoliosis
IBC	In-brace correction
SRS	Scoliosis Research Society
DRU	Distal radioulnar
AV	Apical vertebra
AP	Anteroposterior
